# Range Dynamics of the Moss *Pohlia cruda* in Italy Under Different Climate Change Scenarios

**DOI:** 10.3390/plants14233640

**Published:** 2025-11-28

**Authors:** Giulia Bacilliere, Djordje P. Božović, Marko S. Sabovljević, Marta Puglisi

**Affiliations:** 1 Department of Biological, Geological and Environmental Sciences, University of Catania, Via A. Longo 19, I-95125 Catania, Italy; 2 Institute of Botany and Botanical Garden “Jevremovac”, Faculty of Biology, University of Belgrade, Takovska 43, 11000 Belgrade, Serbia; 3 Department of Plant Biology, Institute of Biology and Ecology, Faculty of Science, Pavol Jozef Šafarik University in Košice, Manesova 23, 040 01 Košice, Slovakia

**Keywords:** bryophyte, range change, species-distribution modeling, ecological niche, global warming

## Abstract

*Pohlia cruda* (Hedw.) Lindb. is a cryophilous moss species with a boreo-arctic montane distribution. As global temperatures continue to rise, high-mountain plant species are increasingly forced to migrate to higher elevations to remain within their ecological and physiological tolerance limits. In this study, we applied ensemble species-distribution modeling (SDM) to evaluate the future niche availability of *P. cruda* in Italy under two greenhouse gas-emission scenarios and two time periods (2050 and 2090). Projections under the intermediate emission scenario (SSP2-4.5) indicate a habitat loss ranging from −24.1% to −46.7%, whereas predictions under the very high emission of greenhouse gases (SSP5-8.5) suggest even greater losses, between −28.1% and −59.9%. These findings point to a substantial reduction, fragmentation, and potential disappearance of suitable habitats for *P. cruda* in the coming decades. This study represents a pioneering application of bryophyte-distribution modeling for the territory of Italy and provides a foundation for integrating such approaches into conservation decisions aimed at preserving biodiversity.

## 1. Introduction

Bryophytes are the second largest group of land plants and are important pioneer organisms. In high mountains, a harsh environment, they are a significant component in fragile ecosystem function and structure (e.g., [1,2]). As ecosystem engineers, they play a pivotal role in developing habitat and microhabitat conditions, and they provide shelters for many other organisms and also other ecosystem services [3]. They are able to retain water, are highly efficient colonisers and stabilisers of bare substrates, preventing erosion or even accumulating microparticles and providing better conditions for other non-pioneering organisms [3]. For instance, soil mosses are significantly and positively associated with increases in the magnitude of carbon sequestration, nutrient cycling, and organic matter decomposition [4].

Lately, we are witnessing the decrease of many populations in high-mountain environments, being unable to shift to higher altitudes. This affects bryophytes as well and increases the risk of extinction not only to them but also to their habitats, decreasing alpine biodiversity rapidly [5].

The evidence that global warming is contributing to the upward shift of species distributions toward higher elevations is well-documented for angiosperms [6], but to some lesser extent for mosses [7]. However, a certain degree of predictive insight may be achieved by projecting the potential current distribution of the species related to the future expected scenarios. This frame highlights the importance of mountains as a conservative refuge for some bryophytes against climate change [8] and highlights the need to preserve the national populations of some species already living on the edge, as well as the national genetic resources.

The modern era demands new approaches to mitigate extinction risks and threats to species survival. One of the tools appearing useful can be species-distribution modeling. This enables not only current distribution and search for suitable habitats of target entity, but also can predict potential distribution range under various climate scenarios and affect the conservation plans. Thus, modeling species distributions has become an important contemporary tool in proper conservation, and it can be seen as an important part of the integrative conservation approach. However, the outcome accuracy can vary among (syn)taxa due to their size and resolution i.e., precision of accessible data. This approach should be constantly developing in an interdisciplinary manner and cannot be a replacement for field data collection, but has advantages with respect to incorporating small data scales into the larger overall picture.

Habitat suitability models are currently widely used as a method to assess and quantify taxa-environment relationships within biodiversity and ecological research spectra [9]. However, there appears to be a lack of analysis focusing on bryophytes as well as on species-distribution modeling at a national scale for Italy.

Thus, we chose as a model a moss *Pohlia cruda* (Hedw.) Lindb., which is well documented recently over Italian territories. An additional argument was that the species is widespread but localised and decreasing in certain areas of its distribution range. This species also occupies a wide ecological niche, and we want to test whether such a model can be less vulnerable to rapid temperature increase and climate change.

*Pohlia cruda* is an acrocarpous moss species with boreo-arctic montane distribution. Its geographic range spans from the Arctic to the Mediterranean area, passing through Madeira and the Canary Islands (La Palma) [10,11]. It is also known to occur in Algeria, Morocco, southern Africa, Asia, Central and South America, Hawaii, Kerguelen Islands, Australia, New Caledonia, and Antarctica [12]. It is present in several protected areas in Europe and classified as Vulnerable (VU) for the Canary Islands and Lithuania, and Near Threatened (NT) for Germany and Northern Ireland [13]. Overall, the European population seems to be outside of the conservation interest areas, and the species is assessed as Least Concern (LC) in Europe [3]. However, the impact of global changes should not be underestimated, especially among its edge populations that already inhabit sites with suboptimal conditions. *P. cruda* can usually be found in dry, shaded crevices and recesses, and beneath overhanging montane cliffs, or on accumulated humus rather than directly on the rock surface, particularly when the rocks are calcareous [14]. Depending on the substrate, *P. cruda* in Italy can be found in association with other species, including *Diplophyllum albicans* (L.) Dumort., *Bartramia pomiformis* Hedw., *Diphyscium foliosum* (Hedw.) D.Mohr, *Mnium hornum* Hedw., and *Pseudotaxiphyllum elegans* (Brid.) Z. Iwats [15]. Furthermore, on moist montane ledges among tall herbs, it occurs in association with mosses such as *Dicranum scoparium* Hedw., *Hylocomium splendens* (Hedw.) Schimp., *Racomitrium ericoides* (Brid.) Brid., and *Rhytidiadelphus loreus* (Hedw.) Warnst. [15,16]. As a moderately acidophytic species, it occurs on slightly acidic substrate in the uplands within phytosociologycal bryophyte communities in association with species that are classified as threatened at the Italian as well as the European level, such as *Mielichhoferia mielichhoferiana* (Funck) Loeske and *M. elongata* (Hoppe & Hornsch. ex Hook.) Hornsch. [17,18].

Recent research conducted in Italy has reported the presence of *P. cruda* within glacial and periglacial habitats of the central-eastern European Alps [19] as well as in volcanic caves located in Sicily [18]. Although both regions belong to the southern distribution edge of the European overall population of *P. cruda*, the Sicilian ones are both the national and European southernmost point known for this species. These two studies show that mountains host a high variety of habitats within the Natura 2000 network where the species is present, such as permanent glaciers (8340), fields of lava and natural excavations (8320), and caves not open to the public (8310) [20]. Some of the species’ habitats, in particular the permanent glaciers (ice cap and glacier—4.2 EUNIS code), are assessed as Vulnerable. Therefore, enhancing research and conservation efforts is crucial to preserving species dependent on them.

With this study, we aim to investigate what could be the potential consequences (such as shifting or habitat loss, or fragmentation) and impacts of climate change in high-mountain habitats related to model moss *P. cruda*, under two possible greenhouse gas-emission scenarios, providing a first overview on a national level for Italy. The results can also implicate habitat- and species-conservation measures and management.

## 2. Results

### 2.1. Ensemble Model Metrics and Variable Importance

**Figure 1:**
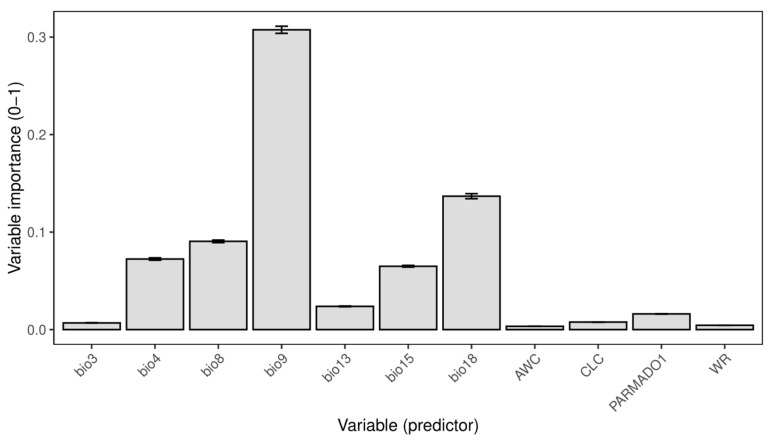
Variable importance of selected variables of the ensemble model. Data are presented as mean ± standard deviation. Bio3—Isothermality; bio4—Temperature Seasonality; bio8—Mean Temperature of Wettest Quarter; bio9—Mean Temperature of Driest Quarter; bio13—Precipitation of Wettest Month; bio15—Precipitation Seasonality; bio18—Precipitation of Warmest Quarter; AWC—Available Water Capacity; CLC—Corine Land Cover; PARMADO1—Parent Material (bedrock); WR—Water Regime.

The total number of computed models amounted to 600, and just those with high predictive power (ROC > 0.85) were selected for the ensemble model created using the mean strategy. The ensemble model had a high predictive power with an ROC value of 0.91 and a TSS value of 0.7. The relative importance of the variables shows that the most important variable among the selected ones is bio9 (Mean Temperature of Driest Quarter) with a value of 0.6 (Figure 1). The following most important environmental predictors are bio18 (Precipitation of Warmest Quarter), bio8 (Mean Temperature of the Wettest Quarter), bio4 (Temperature Seasonality), and bio15 (Precipitation Seasonality) (Figure 1). Out of non-climatic variables, the parent material type (PARMADO1) exhibited the highest variable importance (Figure 1).

### 2.2. Species Response Curves and Ecological Preferences

**Figure 2:**
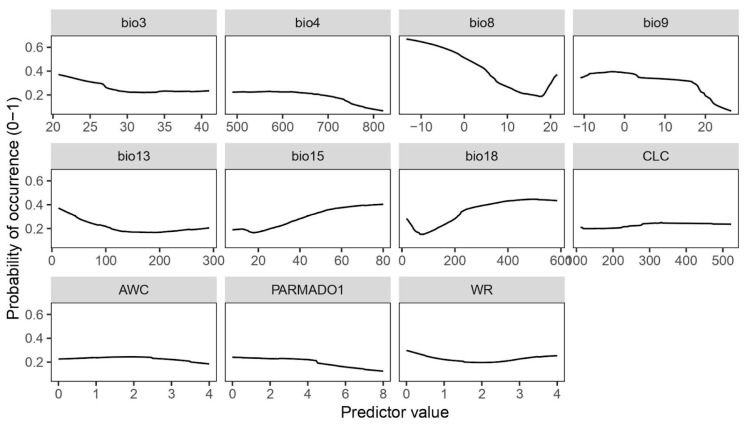
Species response curves generated for the selected mean by ROC algorithm.

Species response curves (Figure 2) provide an insight into the probability of occurrence of the species based on the specific predictor values. In habitats with low temperature and dry environmental conditions, the probability of occurrence of the species increases, as shown in the curve for the mean temperature of the driest quarter (bio9). For the precipitation of the warmest quarter (bio18), the curve shows a positive relation between the species occurrence and the increasing amount of precipitation, indicating that a certain amount of moisture on the substrate is needed during the hot season. Furthermore, a high probability of species occurrence can be expected at low values of the mean temperature of the wettest season (bio8). The response curve obtained for temperature seasonality (bio4) indicates that the species does not tolerate huge fluctuations in temperature during the year. Furthermore, species distribution favours environments with the presence of precipitation seasonality (bio15), as inferred by the bio15 response curve (Figure 2). As suggested by the curve of the amount of precipitation during the wettest month (bio13), high values can reduce the probability of species occurrence. Non-climatic variables such as PARMADO1 underline the parental material where there is a higher probability of occurrence of the moss, such as consolidated-clastic-sedimentary rocks, sedimentary rocks, igneous rocks, and metamorphic rocks. The highest probability of occurrence of the species is observed at the Corine Land Cover categories that correspond to open spaces with little or no vegetation (indicated as 3.3 in the second-level classification), more precisely on bare rocks (indicated with the code 3.3.2). The isothermality (bio3) curve starts to decline for values higher than 25, showing that habitats with a more stable climate (temperature-wise) are more favourable for the species occurrence. The available water capacity (WC) and water regime (WR) values suggest that the substrates where *P. cruda* occurs do not easily retain water.

### 2.3. Species’ Distribution Maps and Range Change

The integration of occurrence data with bioclimatic and non-climatic variables related to lithology and the physico-chemical properties of the soil allowed us to obtain a map of the potential current habitat suitability for the species.

**Figure 3:**
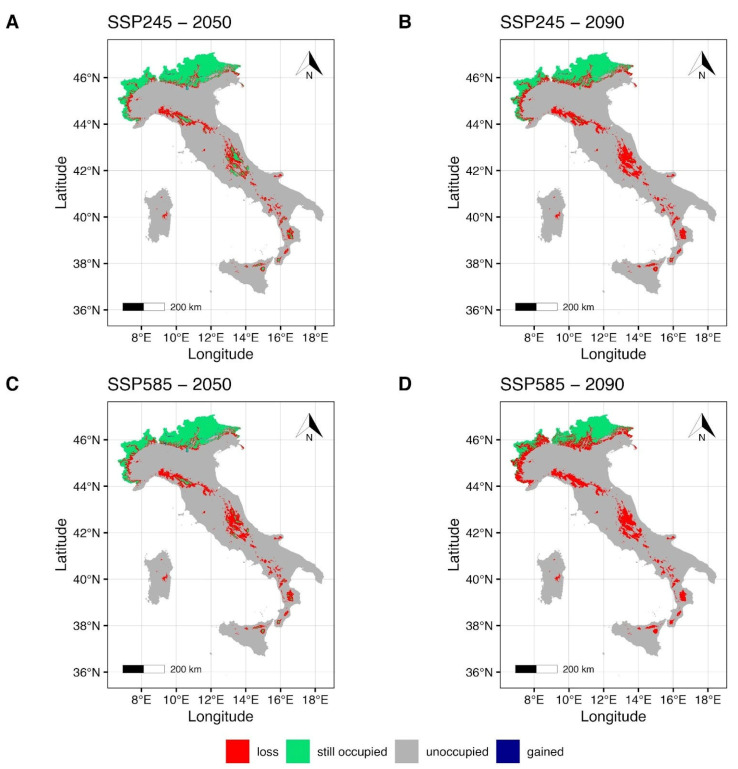
Future projection maps according to MIROC6. Red areas correspond to the lost suitable habitat, green ones to the maintained, and blue ones to the gained surface. References to the year 2050 and ssp2-4.5 are reported in (**A**,**C**). Reference to the year 2090 and ssp5-8.5 are reported in (**B**,**D**).

The MIROC6 climate model maps (Figure 3) under the SSP2-4.5 scenario for the year 2050 (Figure 3A) show a slight reduction in the suitable areas around the Italian Alps in the northern Italian regions Piedmont, Lombardy, Trentino Alto-Adige, and Friuli Venezia-Giulia, with a modest gain in the central-northeastern regions. Central and southern Apennines, together with the Islands (Sicily and Sardinia), show a restriction of the suitable areas (Figure 3A) that are mainly concentrated in the Sibillini mountains, in the Gran Sasso and Maiella massifs (central Italy), and Sila and Aspromonte massifs (southern Italy); all these areas fall within National Parks. Potential suitable areas for Sicily and Sardinia are shown in the northeastern mountains of Sicily, on the Mt Etna (eastern Sicily) and the Gennargentu mountain chain in Sardinia. Estimates for the most pessimistic scenario, SSP5-8.5, for the same period (Figure 3C), indicate a slight increase in gain in the central-northeastern regions of Italy. A notable contraction of the maintained area is evident in the central-north Tosco-Emilian Apennines, and a drastic fragmentation is observed along the central Apennine chain (Figure 3C). Moreover, along the southern Apennines and on the Islands, the potentially suitable environment is limited to some territories of Sila and Aspromonte massifs and to some high-montane areas of Mt Etna.

Forecasts for the year 2090 (Figure 3B,D) under the SSP2-4.5 (Figure 3B) indicate a further decrease in the maintained area in the northern Alps. Moreover, a notable reduction and fragmentation of the maintained area (Figure 3B) is estimated for the central and southern regions of Italy, including the Islands. Specifically, just a few mountain areas within the Tuscan-Emilian Apennines and Sibillini National Park and Gran Sasso e Monti della Laga National Park are shown as suitable for the central Apennines, and a small area in the Aspromonte massif for the south (Figure 3B). Forecasts for the maintained area under the SSP5-8.5 scenario for the year 2090 (Figure 3D) show a reduced but persistent suitable area in the Alpine regions of Italy that spans from the northwest (Piedmont) to the northeast (Trentino Alto-Adige, Friuli Venezia-Giulia), but a complete loss of the maintained areas in the central and southern regions of the country, including the Islands.

**Figure 4:**
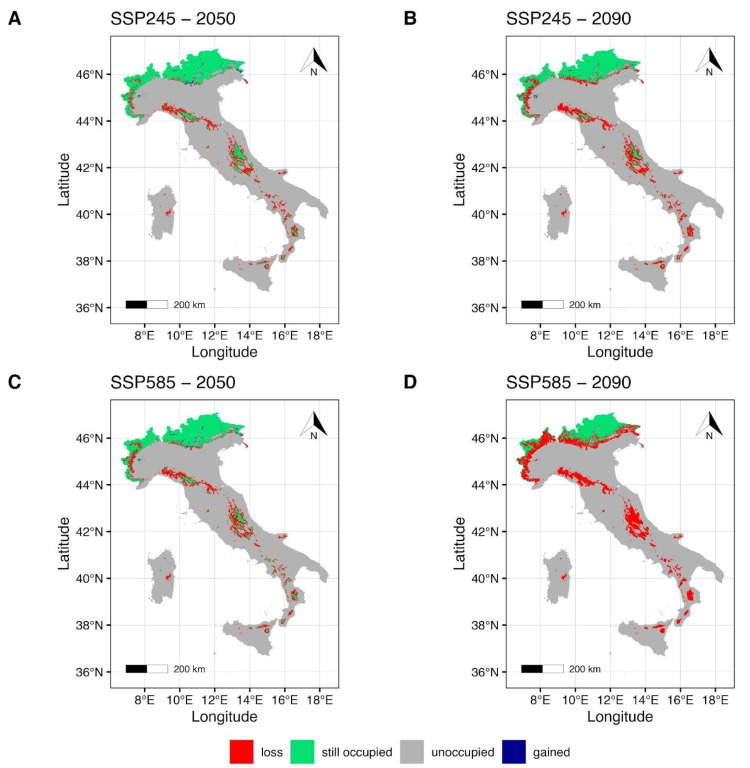
Future projection maps according to EC-Earth3-Veg. Red areas correspond to the lost suitable habitat, green ones to the maintained, and blue ones to the gained surface. References to the year 2050 and ssp2-4.5 are reported in (**A**,**C**). Reference to the year 2090 and ssp5-8.5 are reported in (**B**,**D**).

The EC-Earth3-Veg climate model maps (Figure 4) under SSP2-4.5 for the year 2050 (Figure 4A) show a small reduction in the suitable area in the northern regions of the Italian country, with a weakly pronounced gain from the northeastern to the central alpine regions, and the northwestern region of Piedmont. Some areas of the Apennines, such as the northern Tuscan-Emilian Apennines and the central Apennines, show a reduction and a fragmentation of the suitable area (Figure 4A). Furthermore, the reduction in the suitable area increases in the southern regions of the Italian country, including the Islands (Figure 4A), as the maintained areas appear to be within the southern Apennines (Calabrian Apennines) and in the northeastern mountain areas of Sicily and on Mt Etna. Under the SSP5-8.5 scenario for the year 2050 (Figure 4C), a more evident reduction of the suitable area can be seen, especially for the northwestern and northeastern regions of Italy. Suitable areas in the northern and central mountains of the country, together with the southern Apennines and the Islands, are shown to be drastically reduced (Figure 4C). Forecasts under the SSP2-4.5 scenario for 2090 (Figure 4B) indicate a slightly more pronounced reduction in suitable area and an increase in gained area in the northeastern region of Italy (Piedmont). Significant fragmentation of the suitable area (Figure 4B) is forecast in the central Apennine areas (e.g., Abruzzese Apennines). Furthermore, mountain areas in the southern regions of Italy and on the Islands show a drastic reduction in suitable areas (Figure 4B). Finally, estimates under the SSP5-8.5 scenario for 2090 (Figure 4D) show that suitable areas in the northwestern region of Italy (Piedmont) will be drastically reduced and confined to the highest peaks of the Alpine chain (e.g., Monte Bianco and Cervino). Furthermore, the gained areas previously forecasted under the same emission scenario (Figure 4C) appear to be lost or extensively reduced. Suitable areas in the Central Apennines will be drastically reduced (Figure 4D), leaving a small suitable area confined to the Abruzzo region (Figure 4D). Subsequently, a complete loss of the maintained suitable forecasts for the southern Apennine regions and mountain areas of the Islands (Figure 4D).

**Figure 5:**
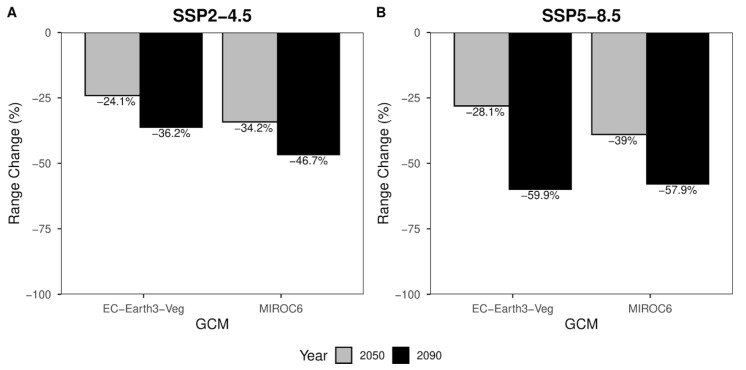
Range change percentage barplot for SSP2-4.5 (**A**) and SSP5-8.5 (**B**). Grey bars represent values for the year 2050. Black bars represent values for the year 2090. Range change values along y-axes are presented at the top of each bar.

Range change percentages (Figure 5) are divided according to shared socio-political pathways. The percentage of habitat loss expected for 2050 (Figure 5A—gray columns) equals −34,2% for MIROC6 and −24,1% for the EC-Earth3-Veg climate models. The same scenario for the reference year 2090 (Figure 5A—black columns) shows a habitat loss of −46.7% and −36.2% for MIROC6 and EC-Earth3-Veg, respectively. For the most pessimistic scenario (Figure 5B), slightly different values of −39% MIROC6 and −28.1% for EC-Earth3-Veg are expected in 2050 (Figure 5B—gray columns). A further decrease in suitable area can be seen in the year 2090 (Figure 5B—black columns), where the loss of suitable area is expected to be −57.9% and −59.9% for MIROC6 and EC-Earth3-Veg climate models, respectively (Figure 5B).

## 3. Discussion

Besides being used to model the current potential and future distribution of organisms [21,22], species-distribution modeling has also been used in the field of conservation biology as an intermediate step in the process of determining species threat status, working on the Extent of Occurrence (EOO) thresholds [23], becoming an important tool in assessing species population trends and threat status [24], especially for threatened species [25]. As a multidisciplinary approach, species-distribution modeling emphasises that numerical techniques support an interesting diversity in applications, arguably with varying degrees of success [26]. However, the choice of one or few algorithms can display differences in the outcomes and lead to different interpretations. Hence, the mechanisms behind each single model must be realistic and well understood [27]. In our study, the use of ensemble models allows taking advantages over all available models that satisfy certain prespecified criteria, producing robust forecasts that can be interpretated [27]. Moreover, recent studies on mosses highlighted the usefulness of species-distribution modelling as a tool to investigate the current habitat suitability of endemic species and to consider the potential consequences of climate change on their peculiar habitat conformity in the future [28]. It is known that correlative species distribution model’s (SDMs) assumptions that species are at equilibrium with the environment [27]. As this kind of research approach avoids other important factors such as biological interactions, estimations relating exclusively to this approach can be too simplistic. Hence, we highlight the importance of a solid knowledge about the chosen species’ biology and ecology.

Our results align with studies related to the impact of bioclimatic variables on the bryophytes’ species distribution [29], highlighting that the precipitation during the warmest quarter is among the most important bioclimatic variables that could determine the extinction risk of the species. Though mosses are poikilohydric plants, water presence is relevant for their life cycle, physiology, and nutrient cycle [30,31]. For instance, precipitation seasonality can be crucial for some moss species, such as in the case of the *Sphagnum* spp. [32]. Furthermore, variables related to temperature were found to be very relevant for the species’ distribution. Indeed, temperature determines the rates of photosynthesis, respiration, and other metabolic processes. As previously confirmed, temperature’s effect can be harmful or lethal, and its effect may differ depending on the hydration status of the plant [33]. The carbon dioxide rise in the atmosphere could increase the photosynthesis rate due to saturation of its target carrier, but only to a small extent, since it will also increase environmental temperature, which will affect key photosynthesis enzymes and cause population decline and vanishing. However, these are some possibilities and additional, antagonistic and/or synergistic effects of many parameters not mentioned here can lead into various destinies, and such consideration should be taken cautiously and further investigated.

Results obtained from the response curves for temperature seasonality and the mean temperature during the wettest quarter align with what is reported for the species’ ecology [34], defining *P. cruda* as a highly cryophytic/frigoriphile species. Moreover, the probability of occurrence of the species increases with low temperatures during the driest quarter. High temperatures can bring significant physiological pressure, such as a decline in photosynthetic apparatus activity and consequently the survival of bryophytes [35]. However, the occurrence of the species at low elevations and under moderate climatic conditions in Central and Northern Europe gives rise to the idea that not all of its European occurrence can be considered cryophilic, from which it can be inferred that there are genotypic variations across Europe.

Non-climatic variables such as available water capacity, water retention, and parent material show a high probability of occurrence on various kinds of substrates that do not retain water for a long period. *Pohlia cruda* is, in fact, also considered a moderate xerophyte [34] that is dry or moist, and mildly acidic to basic habitats [11]. Types of land cover identified as most suitable based on the Corine Land Cover response curve (precisely on bare rocks indicated with the code 3.3.2) correspond to the description of the specific mountain environments provided by Smith et al. [16], identifying them as dry, shaded crevices and recesses of montane rock cliffs.

There is a possibility that the application of terms as dry rocks and wet fissures can be used differently among bryologists across Europe. However, we cannot exclude the ongoing crypto-speciation within *Pohlia cruda*. The differences can be accumulated in genome during longer periods of different phenology among various subpopulations and limited long-distance dispersal events. These, however, require further studies, in the sense of integrative taxonomy, sex expression, and dispersal ability among selected sub-populations in the overall European population.

High-precipitation seasonality and large amounts of precipitation during the warmest quarter outline the need to compensate for high temperature with high humidity, enabling necessary conditions for capsule maturation that usually occur during the warmer months of the year (June–August).

Projected gains for 2050 and 2090 can be explained by the species’ biology and its life cycle. In bryophytes, life forms and life strategies reflect on communities, and the ecological niche they occupy [35,36]. Indeed, *P. cruda* is a pioneer colonist [37] with a prevailing turf life form, and its small spore size enables both short-range and long-range dispersal [36]. However, no vegetative propagules and no evidence on gamete and sporophyte imply life in suboptimal conditions and thus decrease the real species’ potential for dispersal in new and adequate microhabitats, which should also be taken into consideration when predicting species-distribution dynamics. Indeed, gain reduction indicates that dispersal capacity will not be sufficient for boreo-arctic montane bryophytes to cope with climate warming [38]. According to the range change calculations, the no dispersal and full dispersal scenarios had been considered. However, no substantial difference emerged between the two. This suggests that even highly dispersive organisms like bryophytes are not equipped to fully track the rates of ongoing climate change in the next decades [39]. Ongoing global change can drastically alter suitable habitats over time and, as a consequence, compromise the reproductive phenology of the species. Even though the production of phylloids and rhizoidal gemmae under unfavourable conditions can allow a rapid establishment [19], the lack of sexual reproduction with proper pre- and post-zygotic processes would not contribute to the dispersal of species and genetic variation through spore dispersal [39], making the species more vulnerable to climate change.

The difference in the ability of bryophytes from different habitats to track areas of suitable climate suggests that habitat preferences of bryophytes play a leading role in determining current and future species’ ranges. In fact, terricolous and saxicolous bryophytes may show delayed reactions to climate change compared to epiphytes, the latter being more vulnerable to changes in moisture and temperature. However, forest continuity and suitable forest assemblages/vegetation types play a greater role in maintaining epiphytic bryophytes and loss of mature forest ecosystems represents a more important threat to epiphytic bryophytes than climate change [38].

Results obtained from bioclimatic models can serve multiple purposes such as guiding conservation policies, identifying areas where species are likely to occur, or implementing Red List Assessment Categories and applying certain criteria where possible. According to the National Institute of Statistics (ISTAT) [40], about 35% of the whole Italian area is occupied by mountains. In the immediate future (2050), a considerable loss of habitat suitability is estimated. Indeed, this could lead to a reduction in species distribution of almost 39% according to the most pessimistic scenario (MIROC6—ssp8-5.8—2050). Furthermore, the lack of adequate conservation measures could lead to almost double this prediction, reaching nearly 60% habitat loss by 2090 (EC-Earth3-Veg—ssp8-5.8—2090).

Nevertheless, an interesting fact that emerges is a modest gain in terms of surface area, albeit with quite low percentages (Figure 4A,B, blue colour). This result, slightly visible in the maps, shows how the predicted climate changes could lead to habitat fragmentation and altitudinal shifting where possible. It demonstrates that the only conservative environments for the species in the future will be exclusively confined to the Alpine regions of northern Italy (parts of Piedmont, Lombardy, Trentino Alto-Adige, Veneto, and Friuli Venezia-Giulia), leading to the potential extinction of the species in the southern regions of the country. Moreover, it should be considered that some future projections for the years 2050 and 2090 (Figure 4) show that even in the most southern region of Italy (Sicily), habitat suitability is expected to persist at high altitudes of Mount Etna. *P. cruda* has the high sensitivity attributed to heavy metal concentrations (empirically HM value = 0) [41], and therefore the constant emission of potentially toxic elements from the volcano would not allow the species to spread to higher altitudes, even if other combinations of conditions could be suitable.

Moreover, *P. cruda* is also known to be a diagnostic species of some phytosociological associations (*Pohlietum crudae* Privitera and Puglisi 1996 subass. *typicum*) with a punctiform distribution [18]. Hence, losing the species from that environment would lead to an impoverishment of both floristic and coenotic richness and lead to loss, successions, i.e., changes in known phytocenosis.

## 4. Materials and Methods

### 4.1. Study Area and Species-Occurrence Data

The Italian peninsula occupies a surface of about 300,192 km^2^ and is located in a peculiar geographic position within the center of the Mediterranean basin. It has a wide latitudinal extent marked by a conspicuous topographic heterogeneity. Indeed, a significant feature relies on the Alpine and Apennine chain running along the entire peninsula as well as the mountains that can be found in the major Islands (e.g., Punta la Marmora, 1834 m asl—Sardinia; Mt Etna, 3403 m asl—Sicily). The Alpine chain typically characterises the northern part of the country, while central Italy is represented by the presence of 241 peaks over 2000 m asl, dominated by Gran Sasso—Corno Grande at 2912 m asl [42].

Due to this, Italy is characterised by high climatic variability and diversity, representing a long, narrow bridge between the temperate and Mediterranean macrobioclimates [43]. Average rainfall is around 951 mm per year, and updated climate analyses show a significant discrepancy emerging between the north and south of the peninsula. Moreover, while some abnormal excess of precipitation is affecting northern Italy, the south and major islands are suffering from a significant precipitation deficit, with severe and extreme drought conditions, coupled with resulting challenges in water availability.

Species-occurrence data were collected through bibliographical and herbarium research, combined with personal reports noted during fieldwork activities. To avoid outdated information, we set a temporal cutoff using all the available and confirmed data from the year 1968 up to date, obtaining 228 records in total. Further information about the occurrence data is provided as Supplementary Materials. Accounting for the fact that most species-distribution-modeling methods need spatially independent data, spatial bias reduction of the occurrence points was carried out manually using QGIS (v. 4.34.14) [44], selecting one finding per spatial unit of environmental data (30 arc-sec, approximately 1 km^2^ at the equator). Cleaning of the data brought us to 199 final occurrences that were used for the modeling.

### 4.2. Climate Data and Environmental Predictors

In order to fully elaborate on the environmental conditions in which *Pohlia cruda* grows and model its current and future potential habitat suitability, we created a combined dataset of bioclimatic and non-climatic variables (Table 1). The set of bioclimatic predictors corresponds to the 19 bioclimatic variables (bio1–bio19) downloaded from the WorldClim dataset [45] at a spatial resolution of 30 arc seconds. Non-climatic predictors were obtained from the European Soil Database v2.0 [46] and the Corine Land Cover (CLC) dataset [47], both obtained as vector files. In order to match the resolution of the bioclimatic layers, rasterisation of the layers was carried out using QGIS (v. 4.34.14) [44].

The European Soil Database (ESDB) contains a list of Soil Typological Units (STU) that characterise the distinct soil types identified and described. Within the several variables presented in the database, we selected the following ones: Parent Material (PAR-MAT-DOM1) consisting of a list of values from 0 to 9 representing the major group code for the dominant parent material of the STU (e.g., 1 = consolidated-clastic-sedimentary rocks; 2 = sedimentary rocks (chemically precipitated, evaporated, organogenic or biogenic in origin); 3 = igneous rocks; 4 = metamorphic rocks); Available Water Capacity (AWC_TOP) is topsoil available water capacity. This variable is originally represented as a categorical value (L = Low, M= Medium, H = High, VH = Very high, # = no data or not applicable), in order to be used in the model, each letter was substituted with values ranking from 0 to 4 (where 0 = no data or not applicable, 1 = low, 2 = medium, 3 = high, 4 = very high). The water regime (WR or dominant annual average soil water regime) class of the soil profile was set with values from 0 to 4 divided as follows: 0 = no information, 1 = not wet within 80 cm for over 3 months, not wet within 40 cm for over 1 month, 2 = wet within 80 cm for 3 to 6 months, but not wet within 40 cm for over 1 month, 3 = wet within 80 cm for over 6 months, but not wet within 40 cm for over 11 months, 4 = wet within 40 cm depth for over 11 months. The Corine Land Cover (CLC) dataset consists of 44 classes of land-cover/land-use categories at a spatial resolution of 100 m.

With the aim to assess potential changes in the habitat suitability of *P. cruda*, future climate projections were selected within the following Coupled Model Intercomparison Project Phase 6 (CMIP6) global climate models (GCMs): EC-Earth3-Veg [48] and MIROC6 [49]. We obtained the future climate data for the two Shared Socio-economic Pathways (SSPs), the 2-4.5 and 5-8.5 [50], both projected for two different time spans: 2041–2060 (reported here as 2050) and 2081–2100 (reported here as 2090). To match the resolution of current bioclimatic and non-climatic predictors, future bioclimatic variables were downloaded at 30 arc second resolution.

In order to deal with multicollinearity issues among the variables, we implemented the variance inflation factor (VIF) using the vifstep function of the “usdm” R package (v2.1-7) [51] with the VIF threshold set at 10.

### 4.3. Species Distribution Modeling

Habitat Suitability Models (HSM), also referred to as Species-Distribution Models (SDM), are extensively employed to predict potential biodiversity range shifts in response to several future climate change scenarios. As projections by single models can be variable and lead to intermodel variations [52], ensemble models offer a more robust and reliable approach by integrating multiple algorithms, thereby reducing individual model bias and improving the overall predictive accuracy.

The Biomod2 R package [53] has been used as an ensemble platform for species-distribution modeling. It provides several tools to perform species-distribution modeling and it combines single modeling algorithms: ANN—Artificial Neural Network [54], CTA—Classification Tree Analysis [55], FDA—Flexible Discriminant Analysis [56], GAM—Generalised Additive Model [57], GBM—Generalised Boosting Model [58], GLM—Generalised Linear Model [59], MARS—Multiple Adaptive Regression Splines [60], MAXENT—Maximum Entropy [61], MAXNET—Maximum Entropy [62], RF—Random Forest [63], SRE—Surface Range Envelope [64], and XGBOOST—eXtreme Gradient boosting Training [65]. As the models require both presence and absence data, 5 sets of 1000 pseudo-absences were created using a random strategy, as Barbet-Massin [66] advises, because most of the models work with regression methods or machine learning techniques. Each model was run 10 times for each subset of pseudo-absences using 70% of the data for model calibration and 30% of the data for model evaluation. For the creation of an ensemble model, we have selected the individual models with high predictive power (ROC > 0.85). The mean algorithm had been adopted for the production of the ensemble models. Model evaluation was performed using the Relative Operating Characteristic (ROC) and True Skill Statistic (TSS).

Subsequently, the BIOMOD_EnsembleForecasting function was used to project an ensemble model for the quantification and visualisation of the current and future habitat suitability of the species under various climatic scenarios. Final maps used to calculate range change were obtained by using the binary projection rasters.

## 5. Conclusions

With this study, we provide a first pioneering foundation for the application of Species-Distribution Modeling (SDM) in the field of bryological studies for the Italian territory. Our findings reveal that *Pohlia cruda* is likely to experience a marked decline in suitable habitats across Italy under future climate scenarios, with the extent of loss intensifying under higher greenhouse gas concentrations. These projections emphasise the vulnerability of cryophilous, montane bryophytes to ongoing climate warming, which may lead to the fragmentation or disappearance of key refugial areas. The results highlight the urgent need to integrate bryophytes into national and regional conservation strategies, particularly in montane, alpine, and glacial ecosystems.

## Figures and Tables

**Table 1:** List of all variables used in the study, with those selected for modeling based on variance inflation factor (VIF) analysis marked with an asterisk (*). SD—standard deviation; CV—coefficient of variation; ESDAC—European Soil Data Centre; CLMS—Copernicus Land Monitoring Service.

Code	Variable	Unit	Data Source
BIO1	Annual Mean Temperature	°C	WorldClim
BIO2	Mean Diurnal Range	°C	WorldClim
BIO3 *	Isothermality (BIO2/BIO7) (×100)	°C	WorldClim
BIO4 *	Temperature Seasonality	SD	WorldClim
BIO5	Max Temp of Warmest Month	°C	WorldClim
BIO6	Min Temperature of Coldest Month	°C	WorldClim
BIO7	Temp Annual Range (BIO5-BIO6)	°C	WorldClim
BIO8 *	Mean Temperature of Wettest Quarter	°C	WorldClim
BIO9 *	Mean Temperature of Driest Quarter	°C	WorldClim
BIO10	Mean Temperature of Warmest Quarter	°C	WorldClim
BIO11	Mean Temperature of Coldest Quarter	°C	WorldClim
BIO12	Annual Precipitation	mm	WorldClim
BIO13 *	Precipitation of Wettest Month	mm	WorldClim
BIO14	Precipitation of Driest Month	mm	WorldClim
BIO15 *	Precipitation Seasonality	CV	WorldClim
BIO16	Precipitation of Wettest Quarter	mm	WorldClim
BIO17	Precipitation of Driest Quarter	mm	WorldClim
BIO18 *	Precipitation of Warmest Quarter	mm	WorldClim
BIO19	Precipitation of Coldest Quarter	mm	WorldClim
Elevation	Elevation Data	m	WorldClim
PARMADO1 *	Parent Material (PAR-MAT-DOM1)	Categorical	ESDAC
AWC *	Available Water Capacity (AWC_TOP)	Categorical	ESDAC
WR *	Water Regime	Categorical	ESDAC
CLC *	Corine Land Cover	Categorical	CLMS

## Data Availability

All the data are available by authors upon request.

## Supplementary Materials

The following supporting information can be downloaded at: https://www.mdpi.com/article/10.3390/plants14233640/s1, List of the main references used to detect species’ occurrence data used in the paper. As no records have been provided for Basilicata, this region is not present in the list. Moreover, the record provided for the Umbria region was excluded as it’s antecedent to the cut-off defined for this study (1968).
